# Synergistic preparation and application in PCU of α-calcium sulfate hemihydrate whiskers from phosphogypsum and electrolytic manganese residue

**DOI:** 10.1038/s41598-024-56817-5

**Published:** 2024-03-15

**Authors:** Ting Wang, Xuan Ke, Jia Li, Ying Wang, Weiwei Guan, Xia Sha, Chenjing Yang, Tian C. Zhang

**Affiliations:** 1 Key Laboratory of Catalysis Conversion and Energy Materials Chemistry of Education, Engineering Research Center for Heavy Metal Pollution Control of Hubei Province, College of Resources and Environmental Science, South-Central Minzu University, Wuhan, 430074 China; 2 Civil & Environmental Engineering Department, College of Engineering, University of Nebraska-Lincoln, Omaha, NE 68182 USA

**Keywords:** α-CSHWs, Phosphogypsum, Electrolytic manganese residue, Crystal plane, Controlled release, Environmental sciences, Sustainability

## Abstract

The α-calcium sulfate hemihydrate whiskers (α-CSHWs) were first prepared using phosphogypsum (PG) and electrolytic manganese residue (EMR) as raw materials for coating urea, demonstrating excellent controlled-release properties. The effects of different reaction conditions on α-CSHWs, achieved by optimizing the reaction time, the concentrations of NH_4_^+^, Mn^2+^, and other factors, were discussed. Results showed that when the EMR content was 25 wt%, the reaction temperature was 100 °C, and the reaction time was 3 h, α-CSHWs with a length-to-diameter ratio of 39 were obtained. Through experiments and density functional theory (DFT), the mechanism of α-CSHWs preparation was elucidated. The results show that the addition of EMR reduces the content of impurity ions PO_4_^3−^ and F^−^ in PG while introducing NH_4_^+^ and Mn^2+^. Interestingly, both NH_4_^+^ and Mn^2+^ can reduce the nucleation time of α-CSHWs, while PO_4_^3−^, Mn^2+^, and F^−^ are more likely to adsorb on the (0 0 6) crystal plane of α-CSHWs, NH_4_^+^ readily adsorbs on the (4 0 0) crystal plane. The controlled-release performance of modified α-CSHWs incorporated into polyurethane-coated urea (PCU) was investigated, and it was found that the addition of Mα significantly prolonged the nutrient release period, with the period extending up to 116 days for coatings of 5wt% and above. This work not only enhances the efficiency of PG and EMR utilization but also serves as a reference for the straightforward synthesis and application of α-CSHWs.

## Introduction

Phosphogypsum (PG) is an industrial byproduct primarily composed of calcium sulfate dihydrate (DH, CaSO_4_·2H_2_O), generated from the wet process production of phosphoric acid. Currently, the global annual production of PG is approximately 2.8 million tons, and it can be utilized as a soil conditioner, for the production of ammonium sulfate, and as a cement retarder, among other application^1,2^. However, the presence of various impurities in PG, including soluble fluoride (F^-^), soluble phosphates, insoluble fluorides (such as CaF_2_ and Na_2_SiF_6_), insoluble phosphates (such as CaHPO_4_·2H_2_O, Ca_2_(HPO_4_)(SO_4_)·2H_2_O), trace amounts of organic compounds, silica, and trace heavy metals^3–5^, hinders its further resource utilization. Statistics indicate that the recycling rate of PG is only 15%, with a total stockpile exceeding 6 billion tons, leading to increasingly severe environmental issues such as soil and water pollution^6^. Therefore, addressing how to effectively mitigate the impact of impurities on the comprehensive utilization of PG is an urgent and challenging issue.

The preparation of calcium sulfate hemihydrate whiskers (CSHWs, CaSO_4_·0.5H_2_O) using PG as the primary raw material is currently a hot topic and a feasible approach for large-scale treatment of PG^7^. CSHWs, a type of green, high-value-added sub-nanomaterial, are known for their excellent compatibility, low toxicity, and cost-effectiveness, making them widely used in fields such as reinforcement elements and adhesives^8,9^. Depending on the preparation process, CSHWs can exist in two forms: α-CSHWs and β-CSHWs. α-CSHWs are milder in their preparation conditions compared to β-CSHWs, exhibit higher hydration and curing strength, and possess superior mechanical properties^10^. Currently, methods for preparing α-CSHWs include autoclave hydrothermal synthesis, ambient pressure salt solution synthesis^11^, microwave irradiation, and others. Among these methods, microwave irradiation stands out for its ability to rapidly heat and enhance reaction efficiency by replacing traditional heating with microwave radiation^12,13^. For instance, Yan et al.^14^ achieved a conversion rate of 96% for calcium sulfate to α-calcium sulfate hemihydrate after only one hour of microwave irradiation at 100 °C on purified PG and calcium chloride solution, whereas typical reactions for the conversion of dihydrate calcium sulfate to α-CSHWs are carried out at temperatures ranging from 110 to 140 °C. Clearly, microwave irradiation significantly reduces the reaction temperature and time.

When preparing α-CSHWs using industrial gypsum such as PG, it is essential to consider the impact of impurities on the whisker morphology. Impurities in PG, such as soluble fluorides, have a coarsening effect on the α-CSHWs and simultaneously reduce their growth rate^15^. Furthermore, impurities, particularly soluble phosphates, significantly inhibit the formation of α-CSHWs^16^. Therefore, if PG is chosen as the primary raw material for producing α-CSHWs, it is necessary to pretreat the PG, such as by washing or calcination^17,18^. However, these methods often involve high processing costs, the risk of secondary pollution, and are not aligned with sustainable development principles. In recent years, an increasing number of researchers are inclined to utilize solid waste for the co-curing and stable treatment of impurities. For example, Li et al.^19^ effectively stabilized the impurities in PG using tailings, resulting in impurity fluoride ions and heavy metals meeting the required standards. Huang et al.^20^ utilized EMR and PG for cooperative solidification and stabilization without the addition of curing agents but only water. They observed that heavy metals in EMR reacted with excessive phosphate ions and fluoride ions in PG to form phosphates and fluorides, resulting in heavy metal concentrations below the specified limits. Hence, co-curing for the treatment of impurities in PG to purify the production of α-CSHWs could be a potentially viable approach.

Electrolytic manganese residue (EMR) is the waste product left after the production of electrolytic manganese through the acid leaching of rhodochrosite ore and subsequent filtration^21^. As another major solid waste, the stockpile of EMR in China exceeds 150 million tons^22^. Essentially, EMR consists of silicon dioxide, calcium sulfate dihydrate, and contains high concentrations of soluble manganese, ammonia nitrogen, and other heavy metals^23^. Currently, the primary method of handling EMR in China is storage, with a comprehensive utilization rate not exceeding 7%. Storage not only occupies land but also poses a severe ecological threat due to the presence of a large quantity of soluble ions in EMR^24^. Therefore, the urgent need to effectively increase the utilization rate of EMR is paramount.

It is noteworthy that no relevant reports about the collaborative preparation of α-CSHWs using EMR and PG. Particularly, the impact mechanism on whisker morphology remains unclear, in the presence of manganese ions, ammonium ions, etc. Therefore, α-CSHWs were synthesized through microwave-assisted aqueous alcohol method using ball-milled EMR and PG as raw materials. The influence of NH_4_^+^ and Mn^2+^ introduced by EMR on whisker formation and morphology was systematically investigated. The mechanism was elucidated through DFT calculations and various analytical techniques, including XPS. The application of the prepared whiskers, post-modification, in polyurethane-controlled release fertilizer was explored, revealing that modified whiskers exhibit excellent delayed release properties in coated controlled release fertilizers. The aim of this study is to propose a simple and environmentally friendly route for the preparation of α-CSHWs, elucidating its mechanism, and concurrently expanding its potential applications in agriculture.

## Materials and method

### Raw materials

The PG was collected from a phosphate gypsum residue site in Jingmen, Hubei Province, China. The EMR was derived from an electrolytic manganese factory in Guangxi province in China. The chemical compositions of PG and EMR measured by X-ray fluorescence (XRF) are shown in Table 1. The content of calcium sulfate dihydrate in EMR and PG is 51.60% and 83.38%, respectively, as determined by the testing method outlined in Fig. S1 and the standard GB/T 23456-2018. Analytical pure anhydrous ethanol and glycerol were purchased from Sinopharm Chemical Reagent (Beijing, China). Distilled water was used in all processes.

**Table 1:** Chemical compositions of PG and EMR (wt%).

Chemical compositions	SO_3_	CaO	SiO_2_	Al_2_O_3_	P_2_O_5_	Fe_2_O_3_	MgO	MnO	Others
PG	40.099	28.45	9.166	1.568	1.206	0.685	0.222		18.604
EMR	26.733	10.664	18.544	1.985	0.172	5.877	2.882	5.203	27.94

### Preparation of whisker precursor

In this study, 10 g of PG were placed in a ball milling jar with a ball-to-material ratio of 10:1, and the milling was conducted at a rotation speed of 300 rev./min (rpm) for 30 min. Under the same experimental conditions, the composition of the raw materials was altered, with varying relative quantities of EMR to PG, specifically 10 wt% (EMR to 90 wt% PG), 15 wt%, 20 wt%, 25 wt%, and 30 wt%, while maintaining the total material (EMR + PG) in the ball mill at 10 g. Ball milling experiments were performed to obtain precursor whiskers with different proportions. In addition, separate ball milling experiments were carried out by individually adding manganese sulfate monohydrate (ranging from 0.5 wt% to 4 wt%) and ammonium sulfate (ranging from 0.25 wt% to 2 wt%) to PG. These experiments aimed to investigate the roles of Mn^2+^and NH_4_^+^ during the doping process on the whiskers.

### Preparation of α-CSHWs

In this experiment, α-CSHWs were prepared using the atmospheric pressure microwave-assisted aqueous alcohol method. The preparation was carried out using the Anton Paar microwave instrument (MultiWave PRO, Austria), which accommodates 24 reaction vessels, each of which is equipped with temperature and pressure control sensors.

The primary objective of this experiment was to investigate the cooperative treatment of dihydrate calcium sulfate to produce α-CSHWs using EMR and PG in an aqueous alcohol system^25^. Preliminary experiments were conducted to determine reaction temperature, alcohol water ratio, and solid-to-liquid ratio. To initiate the experiment, a solution of glycerol (50% mass concentration) was poured into the reaction vessels. Subsequently, varying proportions of precursor whiskers were added to the solution, maintaining a slurry solid-to-liquid ratio of 1:9. The reaction vessels were then placed in the microwave instrument, and the program was set to a reaction temperature of 100 °C. After a specified duration of reaction (1, 2, 3 h), the slurry was rapidly filtered and rinsed three times with boiling water, followed by a single rinse with anhydrous ethanol. Finally, the samples were collected after drying at 60 °C for 6 h. Furthermore, the slurry (glycerol-water solution) obtained after the initial filtration was collected and reused. The process of synthesizing α-CSHWs was subjected to three parallel experiments.

### Preparation of PCU-Mα

Modified α-CSHWs polyurethane-coated urea (PCU-Mα) was prepared in two steps. The first step involved the preparation of Modified α-CSHWs (Mα). The α-CSHWs selected for this step were obtained from a reaction conducted for 2 h with an addition of 25wt% EMR. At room temperature, 0.6 g of sodium stearate was thoroughly mixed with 50 mL anhydrous ethanol in a conical flask fitted with a stopper. After complete dissolution of sodium stearate, 7.5 g of α-CSHWs were added to the flask. The mixture was stirred (300 rpm) at 90 °C in a constant-temperature water bath for 25 min, followed by hot filtration. The obtained Mα was then dried in a vacuum oven at 60 °C for 12 h.

The second step involved the preparation of Modified α-CSHWs castor oil-based polyurea-coated fertilizers (PCU-Mα). Initially, an appropriate amount of Mα (ranging from 0wt% to 7wt%) was added to 6 g of castor oil (CO). After ultrasonic mixing for 0.5 h, 4 g of polymethylene diisocyanate (PMDI) was added. The mixture was stirred at 300 rpm for 5 min, followed by vacuum defoaming for 15 min in a vacuum oven to obtain uniformly dispersed polyureas containing different contents of Mα (After vacuum defoaming, the samples were dried at 60 °C for 4 h to obtain polyurea films). In a small spray granulator (YC-1000, China), 200 g of urea granules were fluidized at a blower frequency of 45 Hz. Subsequently, 10 g of pure polyurethane containing only CO and PMDI was sprayed onto the surface of the urea granules. After mixing at room temperature for 5 min, the temperature was adjusted to 80 °C and cured for 10 min to form a coating layer. This process was repeated to obtain a second layer of coating. By incorporating polyureas containing different levels of Mα, five different samples were obtained (labeled as PCU, PCU-Mα-1wt%, PCU-Mα-3wt%, PCU-Mα-5wt%, PCU-Mα-7wt%).

### Testing conditions

The concentrations of NH_4_^+^, PO_4_^3−^, Mn^2+^ and F^−^ in whisker precursors and the leaching toxicity of α-CSHWs were determined by the solid waste leaching toxic leaching method (China HJ557–2010). Manganese was measured by an Inductively coupled plasma emission spectrometer (ICP-OES) (ICE 3500 Thermo Fisher, USA). The concentration of ammonia nitrogen was determined by Nessler's Reagent spectrophotometry. Phosphate and fluoride were determined by Ion Chromatography (ICS-2100, USA). The solution pH was measured using an ultra-trace sample volume-type pH electrode (PHS-3C, Shanghai, China). Three parallel experiments were carried out on the contents of NH_4_^+^, PO_4_^3−^, Mn^2+^ and F^−^, and the data in the text are the average of the results of them. The leaching toxicity test results were compared with GB 5085.3-2007 standards, and the findings were presented in Supplementary Table S1.

Calcium sulfate phases were identified by thermogravimetry–differential scanning calorimetry (TG-DSC, STA449F3, Germany) in the temperature range of 25–1200 °C. The identification of the sample of the phase compositions was determined by X-ray diffractometer (XRD, D8 advanced model, Bruker, Germany) using a copper tube radiation source at 40 mA current and 40 kV voltage. The patterns were obtained within 10°–80° and at a scan speed of 10°/min with a Cu Kα radiation source. The chemical compositions of raw materials were investigated by X-ray fluorescence spectrometry (XRF, Zetium, PANalytical B.V., Netherland). The α-CSHWs and Coated fertilizers were subjected to morphology observation by Scanning electron microscopy (SEM, S4800, Hitachi, Japan) at an accelerating voltage of 20 kV and Energy dispersive X-ray spectroscopy system (EDX, Zeiss, Germany). From the SEM images, 50 well-crystallized crystals were selected to measure their length and diameter, which were subsequently measured using Image Pro Plus software. Fourier transform infrared (FTIR) spectra were recorded on an FTIR spectrometer (Nicolet 6700, USA) via the KBr pellet method with a scanning range from 500 to 4000 cm^−1^. The surface elements of α-CSHWs were investigated by X-ray photoelectron spectroscopy (XPS, Escalab250Xi, Thermo Scientific, USA). Water contact angle (WCA) of polyurethane film was determined using a goniometer (OCA50, Dataphysics, Germany).

### Computational method

To elucidate the influence of various factors on the morphology of α-CSHWs within the reaction system, All calculations in this study were performed with the Vienna ab initio Simulation Package (VASP) within the frame of density functional theory (DFT)^26^. Firstly, a 3D model of α-CSHWs was constructed, and the typical crystallographic planes (0 0 6) and (4 0 0) were selected for investigation. The exchange–correlation interactions of electron were described via the generalized gradient approximation (GGA) with PBE functional^27^, and the projector augmented wave (PAW) method was used to describe the interactions of electron and ion^28^. Additionally, the DFT-D3 method was used to account for the long-range van der Waals forces present within the system^29,30^. The Monkhorst–Pack scheme with a 2 × 2 × 1 k-point mesh was used for the integration in the irreducible Brillouin zone^31^. The kinetic energy cut-off of 450 eV was chosen for the plane wave expansion. The lattice parameters and ionic position were fully relaxed, and the total energy was converged within 10^–5^ eV per formula unit. The final forces on all ions are less than 0.02/Å.

Expanding upon the previously described methodology, the adsorption energies of impurity ions, namely F^-^, PO_4_^3–^, Mn^2+^and NH_4_^+^, on the two crystallographic planes of α-CSHWs, (0 0 6), and (4 0 0), were calculated by Eqs. (1), (2), (3), (4), respectively,

1 $${{\text{E}}}_{{\text{ads}}}={{\text{E}}}_{{{\text{F}}}^{-}/{\text{surf}}}-{{\text{E}}}_{{{\text{F}}}^{-}}-{{\text{E}}}_{{\text{surf}}},$$

2 $${{\text{E}}}_{{\text{ads}}}{={\text{E}}}_{{{\text{PO}}}_{4}^{3-}/{\text{surf}}}-{{\text{E}}}_{{{\text{PO}}}_{4}^{3-}}-{{\text{E}}}_{{\text{surf}}},$$

3 $${{\text{E}}}_{{\text{ads}}}={{\text{E}}}_{{{\text{Mn}}}^{2+}/{\text{surf}}}-{{\text{E}}}_{{{\text{Mn}}}^{2+}}-{{\text{E}}}_{{\text{surf}}},$$

4 $${{\text{E}}}_{{\text{ads}}}={{\text{E}}}_{{{\text{NH}}}_{4}^{+}/{\text{surf}}}-{{\text{E}}}_{{{\text{NH}}}_{4}^{+}}-{{\text{E}}}_{{\text{surf}}},$$

where ${{\text{E}}}_{{{\text{F}}}^{-}/{\text{surf}}}$, ${{\text{E}}}_{{{\text{PO}}}_{4}^{3-}/{\text{surf}}}$, ${{\text{E}}}_{{{\text{Mn}}}^{2+}/{\text{surf}}}$ and ${{\text{E}}}_{{{\text{NH}}}_{4}^{+}/{\text{surf}}}$ are the energies of the α-CSHWs surface with adsorbed F^–^, PO_4_^3–^, Mn^2+^and NH_4_^+^, respectively. ${{\text{E}}}_{{{\text{F}}}^{-}}$, ${{\text{E}}}_{{{\text{PO}}}_{4}^{3-}}$, ${{\text{E}}}_{{{\text{Mn}}}^{2+}}$ and ${{\text{E}}}_{{{\text{NH}}}_{4}^{+}}$ represent the energies of the isolated F^–^, PO_4_^3–^, Mn^2+^and NH_4_^+^ ion in a solvent. ${{\text{E}}}_{{\text{surf}}}$ performs the energies of pristine CSH surface.

### Nutrient release characteristics of PCU and PCU-Mα

The nutrient release rate of PCU and PCU-Mα were measured by the static water extraction method at room temperature. Briefly, a total of 10 g of sample was placed in a nylon bag and placed the bag into a glass bottle containing 200 mL of deionized water. The nutrient release rates were measured at 1, 3, 5, 7, 10, 14, 28, 42, 56, and 84 d until the total nutrient released had reached 75%. And the determination method for nitrogen in coated fertilizers involves alkaline potassium persulfate digestion followed by ultraviolet spectrophotometry (China GB11894–89).

## Results and discussion

### XRD pattern of prepared α-CSHWs

**Figure 1:**
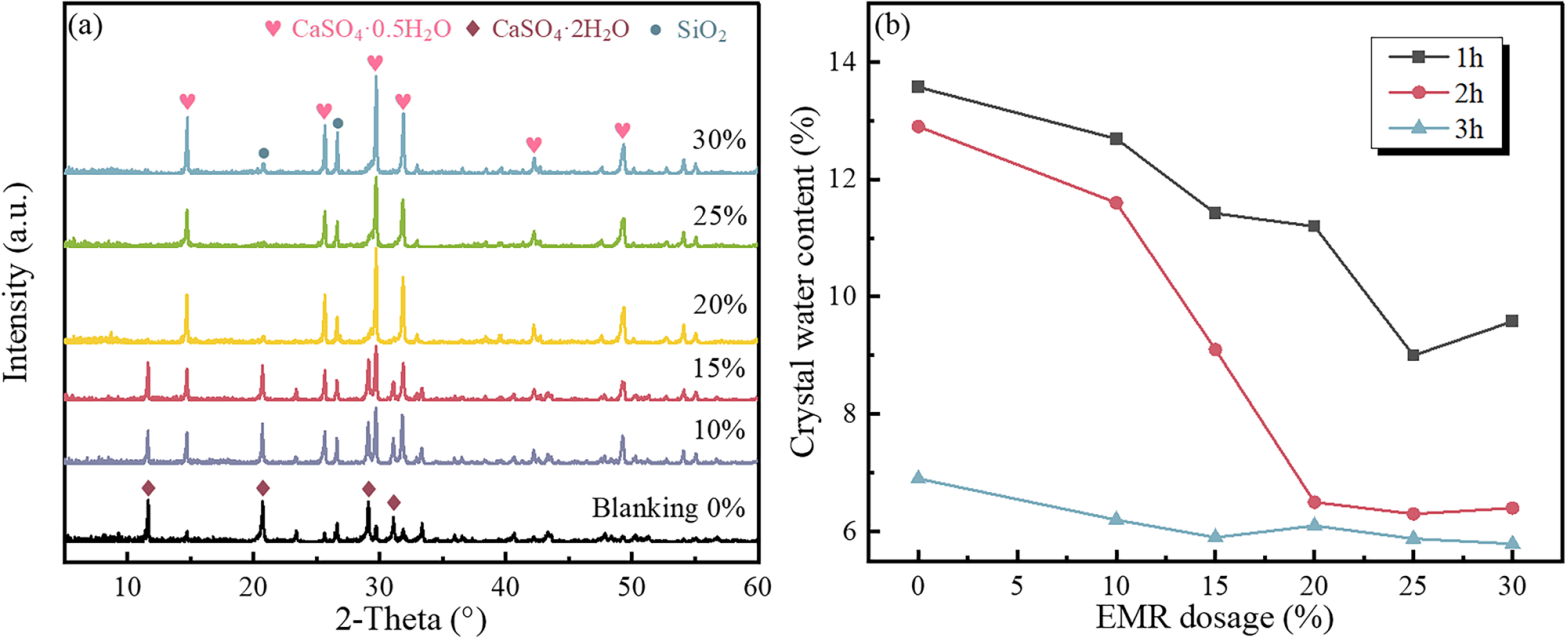
XRD patterns of the samples synthesized by different EMR dosage with different time (**a**) 2 h; (**b**) Evolution of crystal water content of samples.

In order to investigate the impact of EMR addition on the growth of α-CSHWs, single-factor experiments were conducted to optimize and select the reaction time (1 − 3 h) and the quantity of added EMR (0 − 30 wt%). Figures 1a and S3 show that the resulting samples were primarily composed of CaSO_4_·2H_2_O (PDF#01-70-0982), CaSO_4_·0.5H_2_O (PDF#01-81-1849) and SiO_2_ (PDF#01-79-1906). With an increase in reaction time, the diffraction peak intensity of CaSO_4_·2H_2_O gradually decreased. As the quantity of added EMR increased, the conversion of dihydrate calcium sulfate to α-CSHWs progressively increased before reaching a stable level, and the addition of EMR promoted the faster formation of α-CSHWs. Figure 1b shows that with an increase in the quantity of added EMR, the crystalline water content of the prepared samples continuously decreased; after a 2-h reaction, samples with 20 wt% or higher EMR addition exhibited a crystalline water content approaching 6.21% (the theoretical crystalline water content of α-CSHWs).

**Figure 2:**
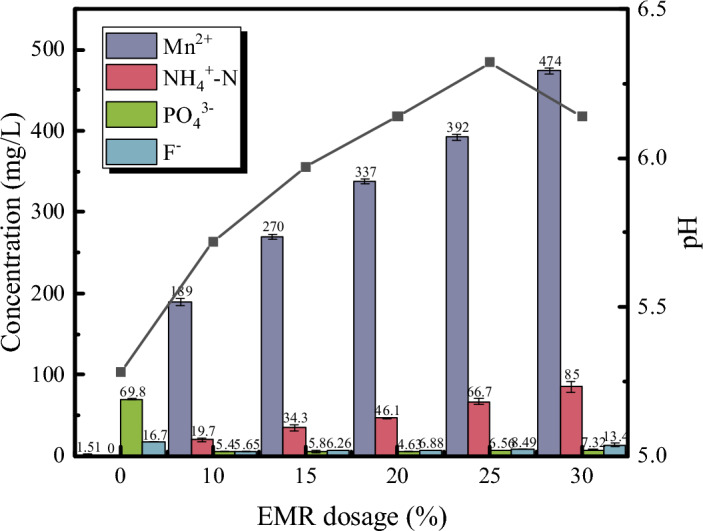
The pH and the concentration of impurity ions with whisker precursor by different EMR dosage.

**Figure 3:**
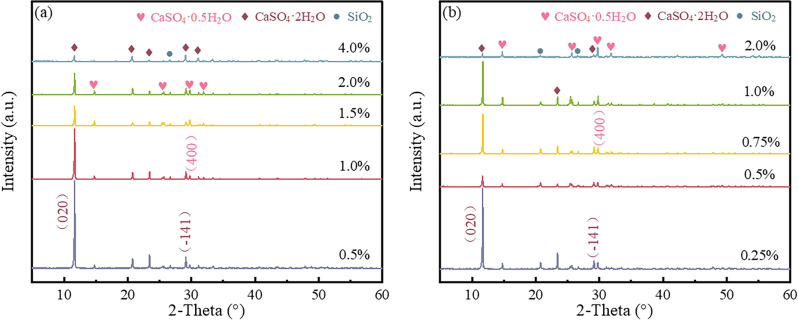
XRD patterns of samples prepared under different reaction conditions: (**a**) the manganese sulfate monohydrate dosage; and (**b**) the ammonium sulfate dosage.

Figure 2 illustrates that with an increase in the amount of EMR added, the pH of the whisker precursor initially increases and then decreases. This might be attributed to a reaction between EMR and PG, resulting in a reduction in soluble phosphate, thereby elevating the pH. However, as the content of EMR surpasses 25wt%, the increased presence of ammonium sulfate carried by the residue leads to a decrease in pH. And from Fig. S1, XRD patterns of whisker precursors with EMR contents ranging from 0 to 30 wt% indicate that F^–^ and PO_4_^3–^ were immobilized and precipitated as MnF_2_, CaF_2_ and Mn_5_(OH)_4_(PO_4_)_2_. Considering that the predominant impurity ions in EMR are Mn^2+^and NH_4_^+^, and referring to the leaching test results shown in Fig. 2, a gradient of Mn^2+^and NH_4_^+^ ion content was established to investigate the impact of EMR added during the preparation of PG on its effectiveness in producing whiskers. Figure 3a shows that with an increase in the content of manganese sulfate monohydrate, the diffraction peak intensity of the (0 2 0) crystallographic plane of CaSO_4_·2H_2_O decreases to some extent. However, when the content exceeds 2.0 wt%, the diffraction peak intensity of the (− 1 4 1) crystallographic plane of CaSO_4_·2H_2_O becomes stronger than that of the (4 0 0) crystallographic plane of CaSO_4_·0.5H_2_O, indicating a reduction, and even inhibition, of the conversion of dihydrate calcium sulfate to hemihydrate calcium sulfate. Figure 3b reflects that with an increase in the ammonium sulfate content, the diffraction peak intensity of the (4 0 0) crystallographic plane of CaSO_4_·0.5H_2_O is enhanced relative to the peak of the (− 1 4 1) crystallographic plane of CaSO_4_·2H_2_O, suggesting a promotional effect of NH_4_^+^ on the preparation of α-CSHWs.

### Morphology of prepared α-CSHWs

**Figure 4:**
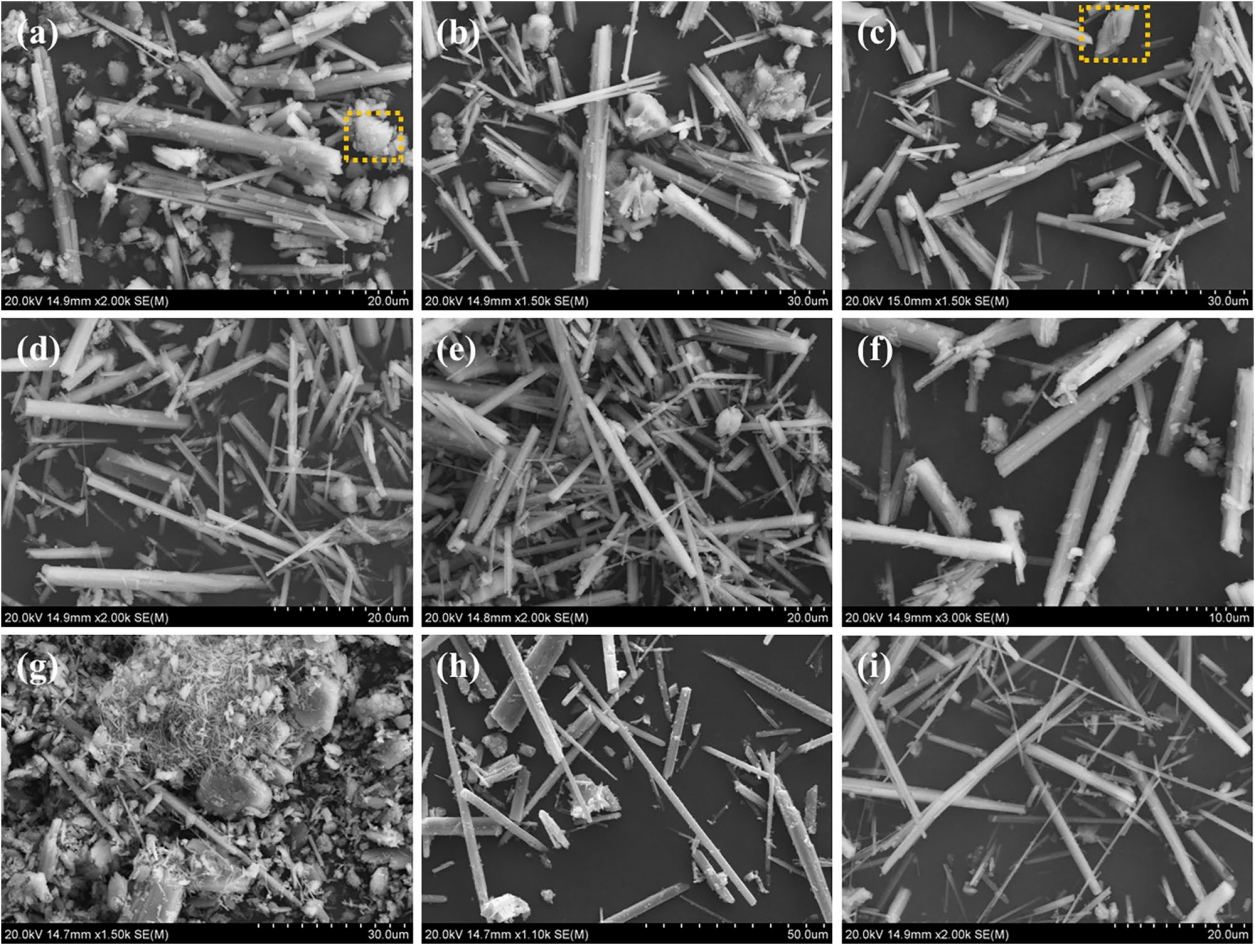
SEM images of the α-CSHWs synthesized in 2 h with different EMR dosage (%): (**a**) 0, (**b**) 10, (**c**) 15, (**d**) 20, (**e**) 25, and (**f**) 30%. SEM images of (**g**) 4% manganese sulfate monohydrate, (**h**) 2% ammonium sulfate, and (**i**) 25% EMR in 3 h.

Figure 4 reveals clearly visible fibrous crystalline structures. Figure 4a–c show that samples obtained with EMR additions of 0–15 wt% after a 2-h reaction exhibit distinct blocky structures. Typically, the transformation from dihydrate calcium sulfate to α-CSHWs is considered a process of dissolution and recrystallization. Combined with XRD analysis results, the blocky structures in these samples can be identified as dihydrate calcium sulfate. Figure 4d–f show that the morphology of whiskers produced with 20 wt% and 25 wt% EMR content is similar. However, when the EMR content is 30 wt%, the diameter of α-CSHWs crystals increases, displaying visible coarse columnar structures. This may be attributed to the high manganese content exceeding 2 wt% in the whisker precursor, which exerts an inhibitory effect on the longitudinal growth of the whiskers. For samples with 30 wt% EMR content after a 2-h reaction, SEM–EDS testing was conducted, and the results are presented in Fig. S2. The microblocky structures were determined to be SiO_2_ rather than dihydrate calcium sulfate using surface scanning.

Figure 4g demonstrates that the introduction of 4 wt% manganese sulfate monohydrate in the whisker precursor inhibits the transformation of dihydrate calcium sulfate into α-CSHWs, consequently restraining the generation of α-CSHWs. In Fig. 4h, introducing 2 wt% ammonium sulfate into PG with a 2-h reaction time results in larger aspect ratios of α-CSHWs crystals, indicating that NH_4_^+^ promotes the formation and longitudinal growth of α-CSHWs. This is consistent with the influence of Mn^2+^and NH_4_^+^ on the formation of α-CSHWs observed in XRD. Figure 4i shows that after 3 h, whiskers produced with a 25 wt% EMR content can achieve an aspect ratio of 39, indicating that the whiskers are still growing.

**Figure 5:**
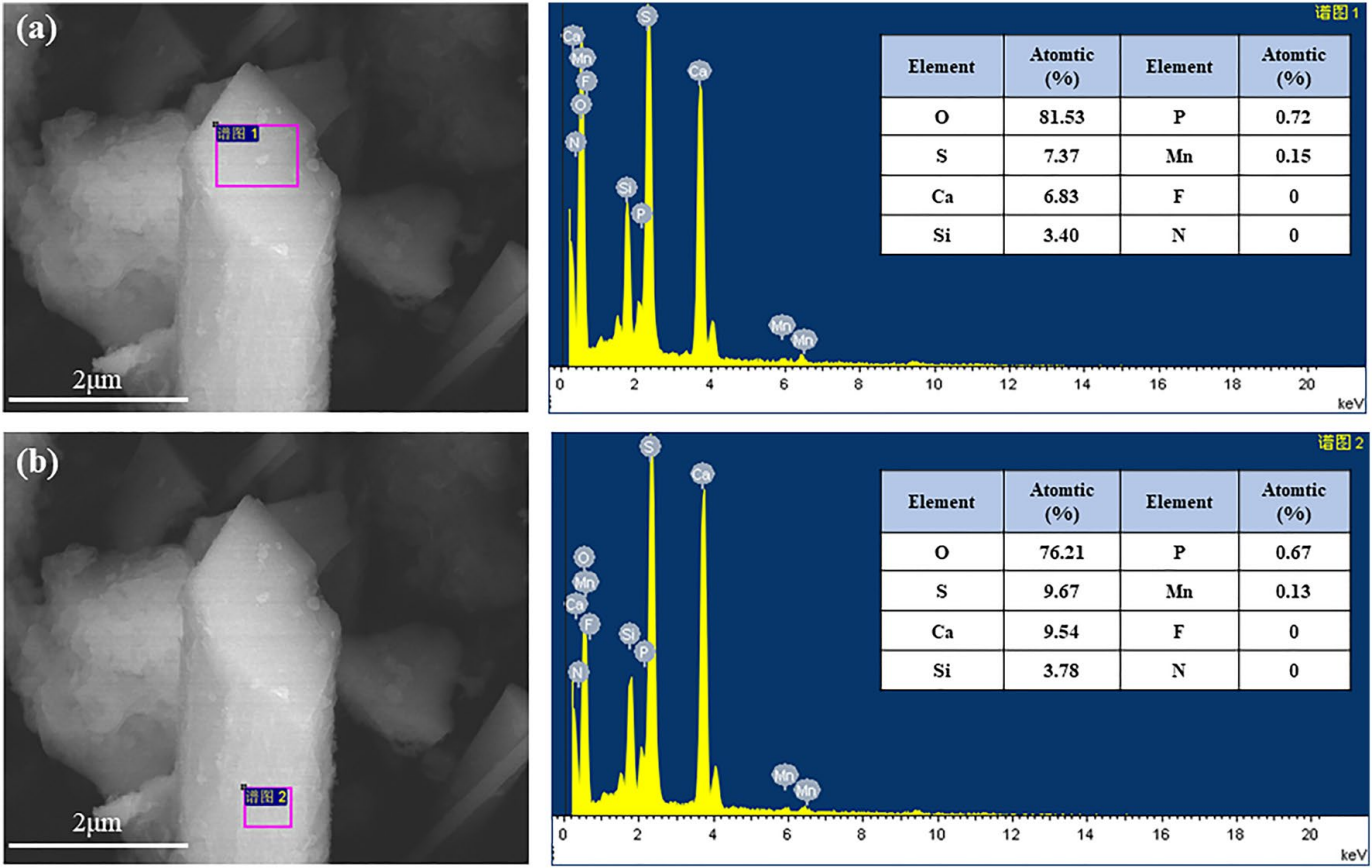
SEM–EDS images of the α-CSHWs synthesized in three h with 30% EMR dosage: (**a**) top, and (**b**) side surface of the whisker.

Figure 5 shows that the manganese content and the phosphorus content on the top surface of the whiskers are higher than that on the side surface. Additionally, no detectable levels of fluorine (F) or nitrogen (N) elements were found on both the top and side surfaces. This phenomenon may be attributed to the facile substitution of calcium by manganese on the top surface and the adsorption of phosphate ions (PO_4_^3–^) on the top surface of α-CSHWs through binding with Ca^2+^. The NH_4_^+^ ions, in conjunction with SO_4_^2–^, form ion pairs and adsorb on the negatively charged side surface. These NH_4_^+^ ions can also decouple into free NH_4_^+^ ions, which is why no N elements were detected on the surface of α-CSHWs. The absence of detectable fluorine elements may be due to the low concentration of F^–^ ions.

### TG-DSC

**Figure 6:**
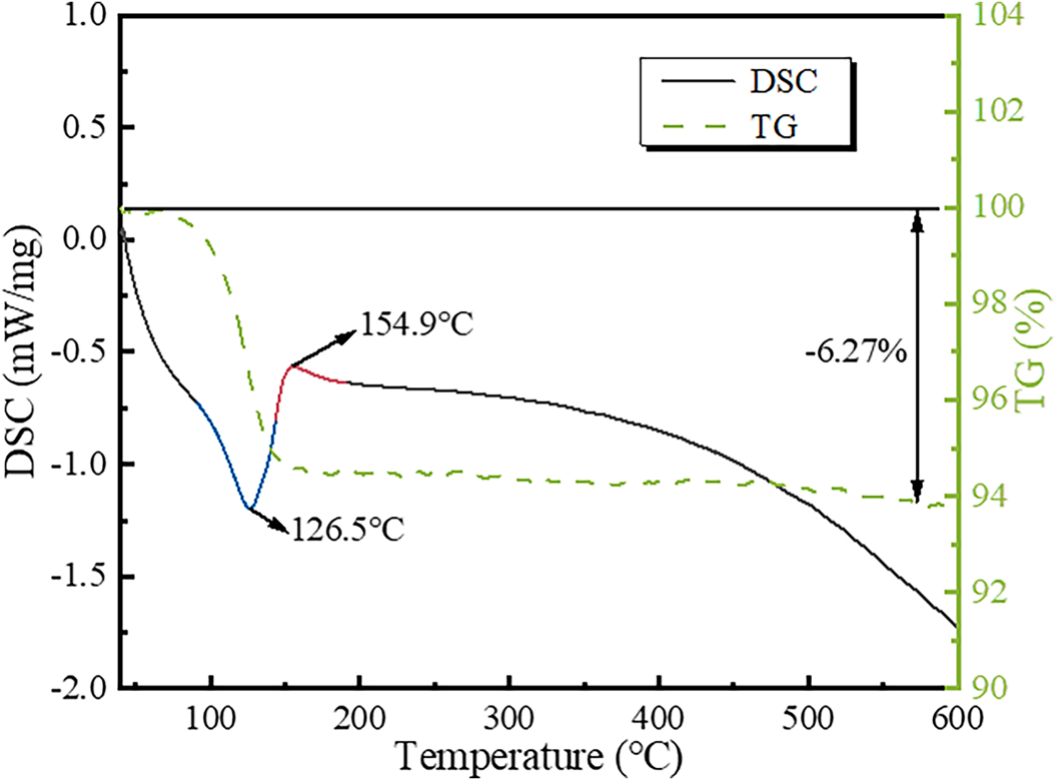
TG-DSC of the α-CSHWs synthesized in three hours with 25% EMR dosage.

As both crystalline forms of calcium sulfate hemihydrate whiskers (α-CSHWs and β-CSHWs) can potentially coexist in the alcohol-water system, TG-DSC analysis can effectively distinguish between them. The TG curve in Fig. 6 reveals a weight loss of 6.27% in the temperature range of 40 to 600 °C, which closely matches the theoretical value of crystalline water content for α-CSHWs (6.21%)^32,33^. The DSC curve displays an endothermic peak at 126.5 °C and an exothermic peak at 154.9 °C^34^, indicating the obtained sample is α-CSHWs rather than β-CSHWs (as β-CSHWs exhibit an exothermic peak around 350 °C)^35^.

### FT-IR analysis

**Figure 7:**
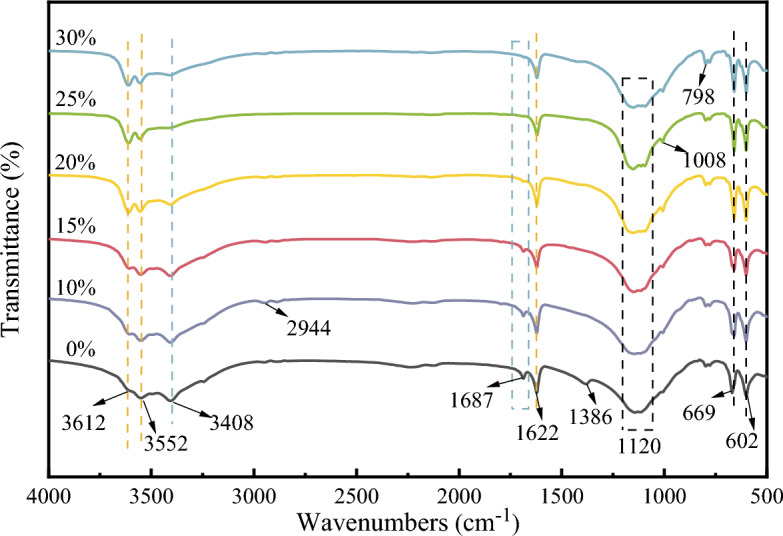
FTIR spectra of the samples synthesized in two hours with different EMR dosage.

Figure 7 shows that the peaks at 3612 and 3552 cm^–1^ correspond to the stretching vibrations of crystalline water –OH groups within α-CSHWs, with the 1622 cm^–1^ peak attributed to the bending vibration of α-CSHWs’ crystalline water^36,37^. The absorption peaks at 669 and 602 cm^–1^ originate from the bending vibrations of SO_4_^2–^ ions within α-CSHWs, while the broader peak at 1120 cm^–1^ corresponds to the stretching vibration of SO_4_^2–^ ions^38^. The absorption peak at 3408 cm^–1^ is attributed to the adsorbed free water –OH on the whisker surface, and the peak at 1687 cm^–1^ is due to H–O–H bending vibrations^39^. These observations indicate that an increased EMR content leads to a reduced presence of dihydrate calcium sulfate after a 2-h reaction, favoring α-CSHWs formation. Moreover, the peaks at 798 cm^–1^ and 1008 cm^–1^ are attributed to the symmetric stretching vibrations of Si–O–Si, in agreement with the presence of SiO_2_ detected through XRD analysis^40,41^. The emergence of new absorption peaks at 2944 cm^–1^ and 1386 cm^–1^ may be linked to the stretching vibrations of –CH_3_ and –CH_2_ groups^42–44^. Suggesting the involvement of glycerol in the formation reaction of α-CSHWs, as these peaks persist in the samples even after multiple washes^45^.

### XPS analysis

**Figure 8:**
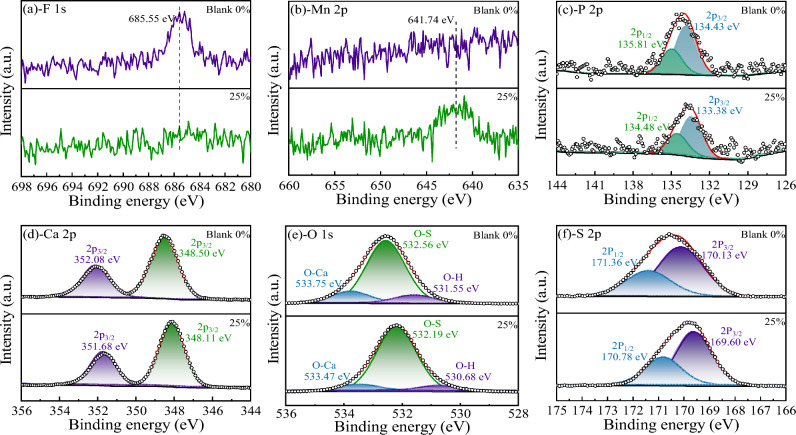
XPS spectra of the α-CSHWs synthesized with 0% and 25% EMR dosage: (**a**) F 1 s, (**b**) Mn 2p, (**c**) P 2p, (**d**) Ca 2p, (**e**) O 1 s, and (**f**) S 2p.

XPS was employed to investigate the interactions between impurity ions (F^–^, PO_4_^3–^, Mn^2+^and NH_4_^+^) and the surface of α-CSHWs crystals. All other elements were core-level corrected to 284.8 eV using the C1s peak as a reference. Combined with the relative elemental content of whisker surfaces shown in Table 2, Fig. 8a–c demonstrate that when 25 wt% EMR is added, the synthesized α-CSHWs exhibit no detectable XPS peaks for F. Furthermore, the P XPS peak intensity significantly decreases compared to the α-CSHWs prepared using pure PG, and Mn elements can be distinctly detected. This corresponds to the reduction in F^-^ and PO_4_^3–^ content in the whisker precursor after EMR addition, as shown in Fig. 2.

**Table 2:** Surface atomic concentration of the α-CSHWs crystals prepared with different EMR dosage.

EMR dosage	S 2p	Ca 2p	O 1s	Si 1s	P 2p	Mn 2p	F 1s	C 1s	K 2p
0%	10.42	9.74	61.87	3.66	0.74	0	0.54	12.07	0.39
25%	11.72	10.66	62.50	3.72	0.55	0.15	0	9.94	0.28

Figure 8d–f show that in whiskers prepared with 25 wt% EMR, the binding energies of Ca 2p, O 1 s, and P 2p decrease by 0.39, 0.37, and 0.53 eV, respectively. The peaks at around 533 eV and 532 eV in O 1 s are attributed to Ca–O and S–O bonds, while the peak at around 531 eV is attributed to H–O bonds^46^. Impurity elements such as F and P act as strong electron-absorbing groups, which absorb X-ray-excited photoelectrons, resulting in a shielding effect and causing an increase in the binding energy of elements like O. Therefore, both Fig. 8 and Table 2 indicate a reduction in impurity elements in whiskers prepared with 25 wt% EMR compared to whiskers prepared with pure PG.

### Adsorption of impurities on α-CSHWs surface: DFT calculation

#### The impact of glycerol on whisker formation

**Figure 9:**
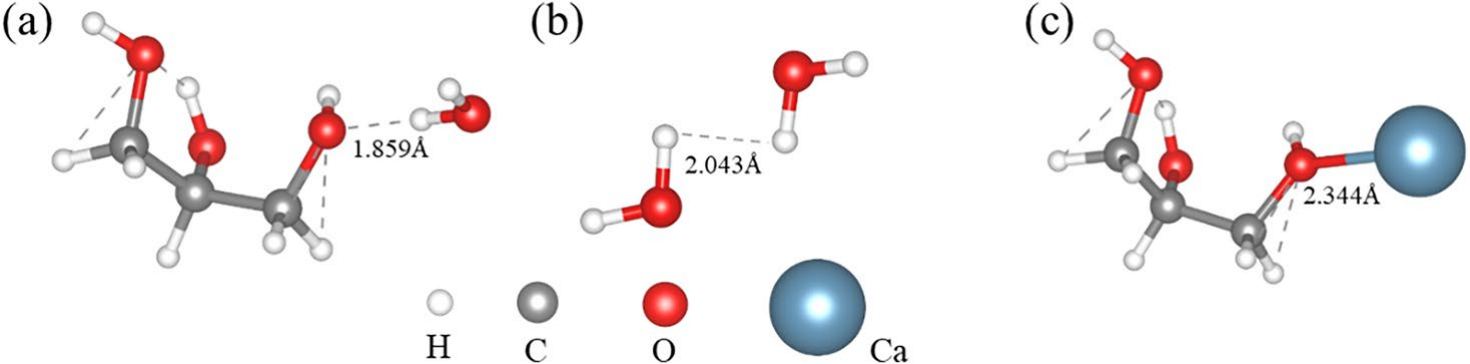
Different combinations in a glycerol and water system: (**a**) glycerol-water, (**b**) water-water, and (**c**) glycerol-Ca.

In the case of the alcohol water system, the binding configurations between glycerol and water, as well as glycerol and calcium atoms, are illustrated in Fig. 9, and the binding energies are presented in Table 3. DFT results reveal the formation of hydrogen bonds between glycerol and water. The binding energy between glycerol and water is − 0.28 eV, which is lower than the binding energy of water with water (− 0.20 eV). This suggests that the hydrogen bonds between alcohol and water are stronger than those between water molecules, effectively reducing the water activity and promoting the transformation of dihydrate calcium sulfate into α-CSHWs. The binding energy between glycerol and calcium atoms is − 0.57 eV, indicating that glycerol readily binds to calcium, facilitating the dissolution of dihydrate calcium sulfate. Moreover, calcium bound to glycerol can also participate in the growth of α-CSHWs, consistent with the stretching vibrations of -CH_3_ and -CH_2_ groups detected in the FT-IR spectra.

**Table 3:** Binding energy in a glycerol and water system calculated by DFT.

	Binding site	E_bin_ (eV)	Distance (Å)
G-W	O–H	− 0.28	1.859
W-W	H–H	− 0.20	2.043
G-Ca	O-Ca	− 0.57	2.344

#### The impact of various impurity ions on whisker formation

**Figure 10:**
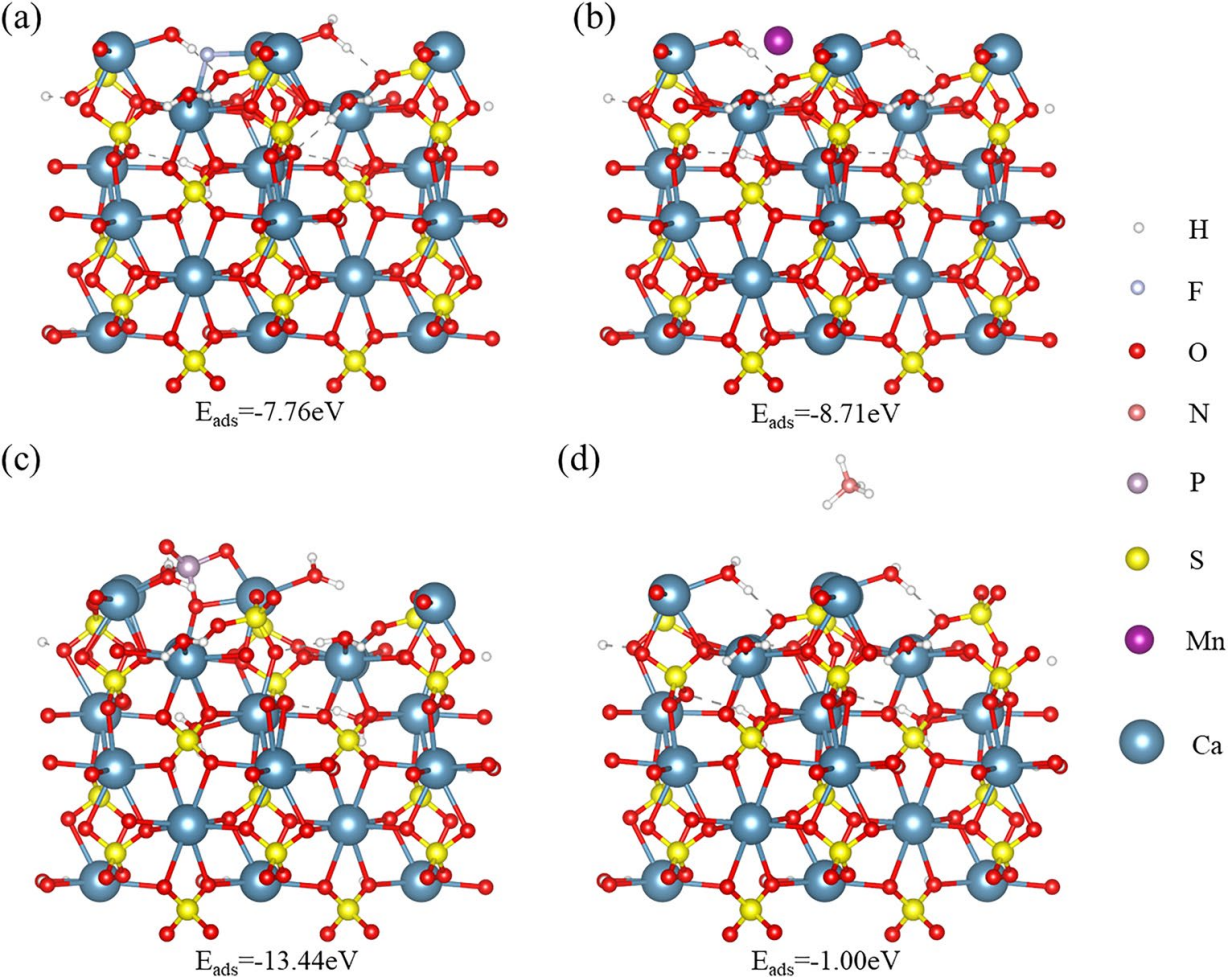
Adsorption configurations of different impurity ions on α-CSHWs (0 0 6) surface: (**a**) F^–^, (**b**) Mn^2+^, (**c**)PO_4_^3–^ and (**d**) NH_4_^+^.

**Figure 11:**
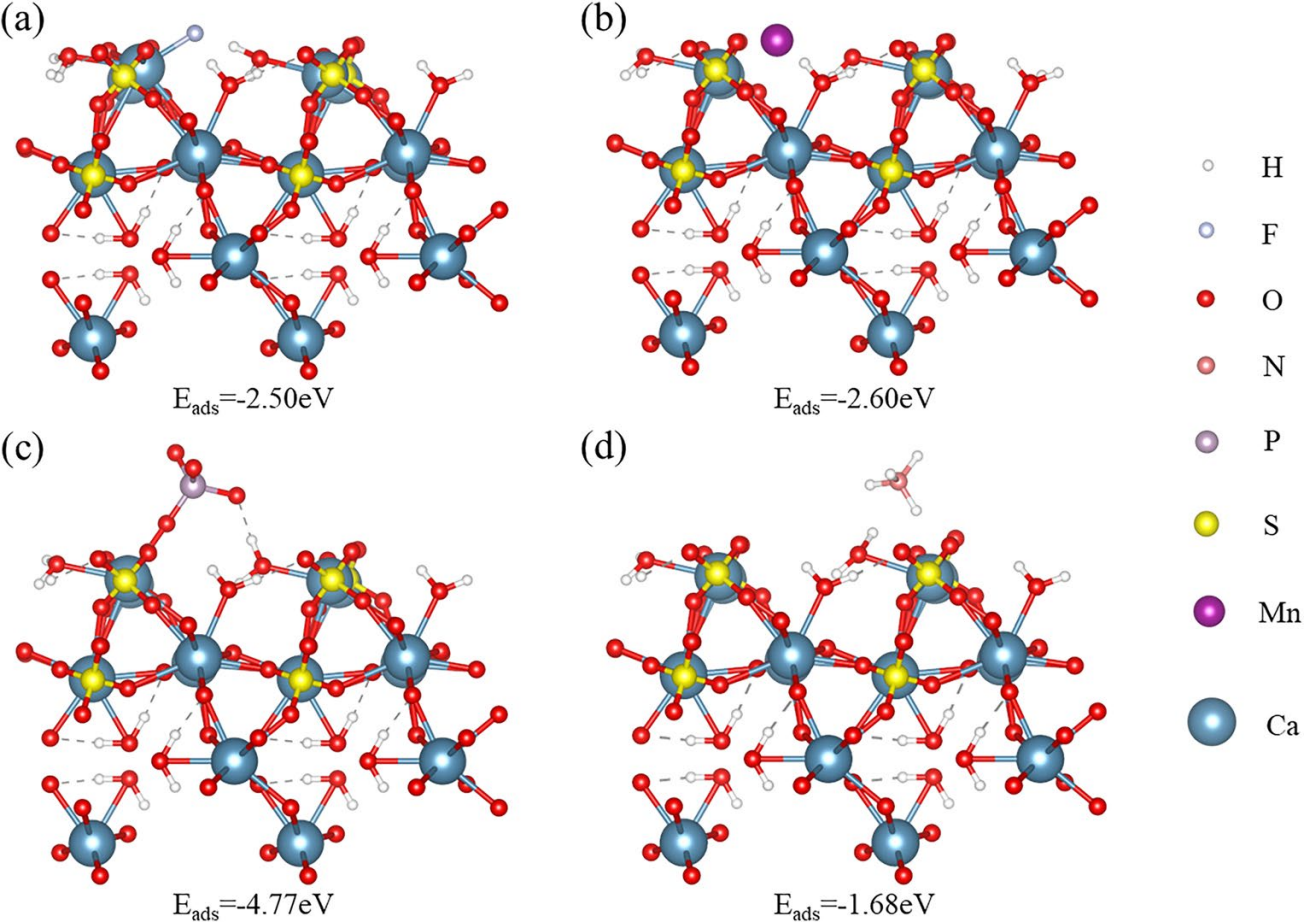
Adsorption configurations of different impurity ions on α-CSHWs (4 0 0) surface: (**a**) F^–^, (**b**) Mn^2+^, (**c**) PO_4_^3–^, and (**d**) NH_4_^+^.

The typical crystallographic planes of α-CSHWs, including (2 0 0), (3 1 0), (4 0 0), (1 1 4), and (0 0 6), were identified through XRD analysis. For this study, the (0 0 6) and (4 0 0) planes, representing the top and side surfaces of α-CSHWs, were selected for investigation. As shown in Figs. 10 and 11, when F^-^ ions adsorb onto the surface of α-CSHWs, their adsorption energies on the (0 0 6) and (4 0 0) crystal planes are − 7.76 eV and − 2.50 eV, respectively. This indicates that F^-^ ions exhibit stronger adsorption on the (0 0 6) plane of α-CSHWs, binding with Ca^2+^ ions on α-CSHWs' surface, thereby impeding top surface growth and consequently inhibiting whisker longitudinal growth. Anionic PO_4_^3–^ ions exhibit adsorption energies of − 13.44 eV on the (0 0 6) plane and − 4.77 eV on the (4 0 0) plane. Cations Mn^2+^ and NH_4_^+^ exhibit adsorption energies of − 8.71 eV and − 1.00 eV on the (0 0 6) plane, respectively, and − 2.60 eV and − 1.68 eV on the (4 0 0) plane, respectively. This indicates that similarly to PO_4_^3–^, Mn^2+^, and F^-^, their stronger binding on the (0 0 6) crystal plane hinders the longitudinal growth of the whiskers. In contrast, NH_4_^+^ with higher adsorption energy on the (4 0 0) plane partly inhibits the radial growth of the whiskers.

In summary, the four types of impurity ions exert varying effects on whisker growth. F^–^, PO_4_^3–^, and Mn^2+^ are more prone to adsorb on the whisker's top surface, thus hindering longitudinal whisker growth, whereas NH_4_^+^, by comparison, tends to adsorb on the whisker's side surface, promoting growth in the radial direction.

### α-CSHWs formation mechanism and performance

In an alcohol-water system, the conversion of dihydrate calcium sulfate to α-CSHWs is considered a process of dissolution followed by re-nucleation and growth, with the reaction proceeding as follows:

5 $${\text{2CaSO}}_{{4}} \cdot{\text{2H}}_{{2}} {\text{O }} + {\text{ M}}^{{{\text{b}} + }} \left( {{\text{aq}}} \right) \Leftrightarrow {\text{2Ca}}^{{{2} + }} \left( {{\text{aq}}} \right) + {\text{ SO}}_{{4}}^{{{2} - }} \left( {{\text{aq}}} \right) + \, \left[ {{\text{MSO}}_{{4}} } \right]^{{\left( {{2} - {\text{b}}} \right) - }} + {\text{ 4H}}_{{2}} {\text{O }}\left( {\text{l}} \right),$$

6 $${\text{2Ca}}^{{{2} + }} \left( {{\text{aq}}} \right) \, + {\text{SO}}_{{4}}^{{{2} - }} \left( {{\text{aq}}} \right) \, + \, \left[ {{\text{MSO}}_{{4}} } \right]^{{\left( {{2} - {\text{b}}} \right) - }} + {\text{ H}}_{{2}} {\text{O }}\left( {\text{l}} \right) \Leftrightarrow {\text{ 2CaSO}}_{{4}} \cdot0.{\text{5H}}_{{2}} {\text{O}} + {\text{ M}}^{{{\text{b}} + }} \left( {{\text{aq}}} \right).$$

**Figure 12:**
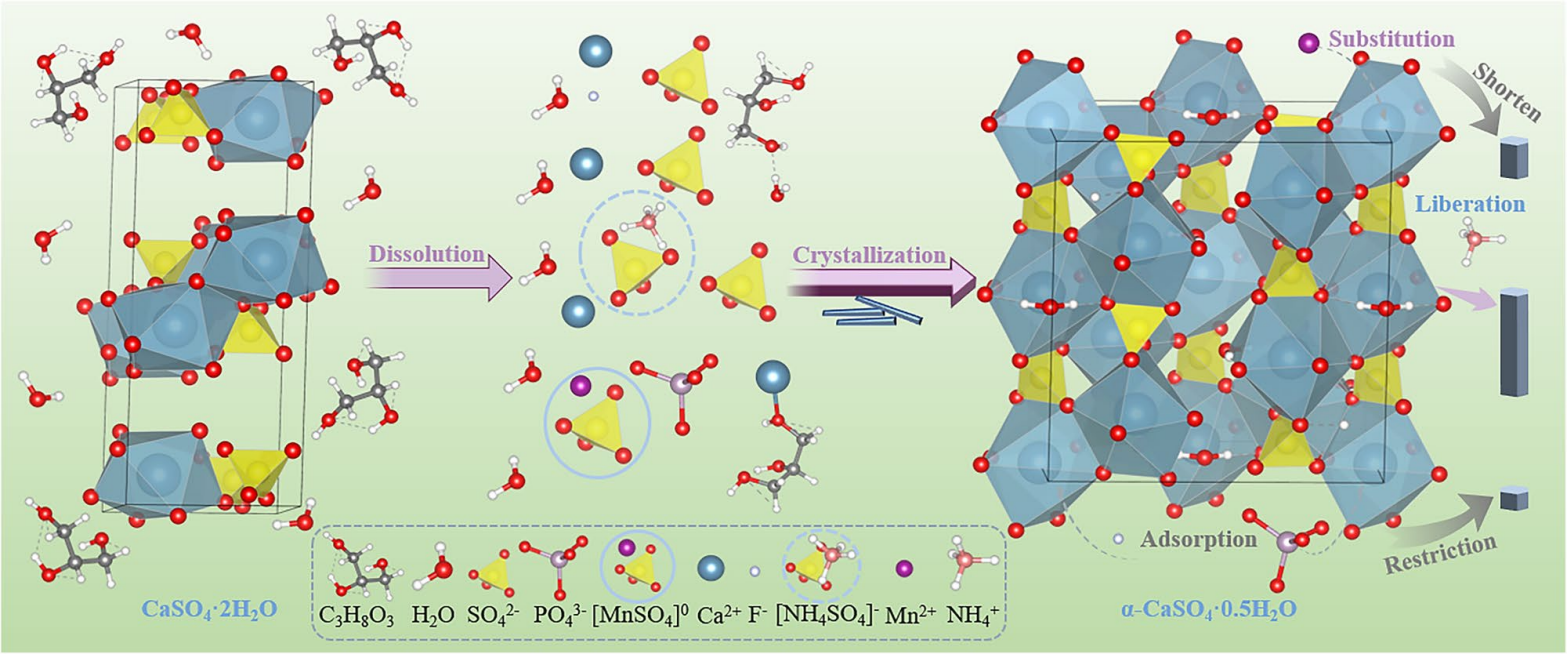
α-CSHWs formation mechanism in the glycerol and water system with EMR doping.

As shown in Fig. 12, during the dissolution phase of dihydrate calcium sulfate, glycerol in the system forms associations with water, reducing the water activity and promoting the dissolution of dihydrate calcium sulfate. When an electrolytic manganese slag is added, Mn^2+^ and NH_4_^+^ are introduced, forming [MSO_4_]^(2–b)–^ ion pairs^47^. This further increases the concentrations of Ca^2+^ and SO_4_^2–^ in the solution, raising the system's supersaturation, thus providing conditions for the nucleation of α-CSHWs and shortening the nucleation time. Most [MSO_4_]^(2–b)–^ ion pairs in the solution replace free SO_4_^2–^ as the primary reactant for α-CSHWs nucleation. Due to the strong decoupling capability of [NH_4_SO_4_]^–^, the SO_4_^2–^ carried by the ion pairs decouples at the nucleation sites for α-CSHWs and free NH_4_^+^ promotes whisker growth. [MnSO_4_]^(0)^, being less prone to decoupling, readily restrict further whisker growth after substituting calcium to form whiskers during the nucleation stage. On the other hand, F^–^ and PO_4_^3–^ in the solution tend to adsorb on the whisker's top surface, leading to radial whisker growth.

For the preparation of α-CSHWs, attention is typically directed towards the aspect ratio of the whiskers, with higher aspect ratios indicating greater potential utility. Table 4 presents the aspect ratios of calcium sulfate hemihydrate whiskers prepared under different reaction conditions. It is noteworthy that in this study, there was no pre-treatment such as washing or acid treatment, and the whiskers were generated with a high aspect ratio of up to 39 at relatively low reaction temperatures and short reaction times. Additionally, the glycerol solution in the reaction system does not cause additional damage to the vessel. Therefore, the environmentally friendly, simple preparation process, high efficiency, and excellent performance make the microwave-assisted aqueous alcohol method with ball-milled EMR and PG a promising method for the preparation of α-CSHWs.

**Table 4:** Performance comparison of various methods for preparing calcium sulfate hemihydrate whiskers.

Raw material	Preparation method	Temperature (℃)	Time (h)	Average aspect ratio	References
Calcified jarosite sediment	Hydrothermal conditions	140	6	10–60	Tan et al.^48^
Acid-washed PG	Alcohol-water hydrothermal	110	2	30.99	Yang et al.^49^
PG	Hydrothermal conditions	130	6	28	Yang et al.^50^
Washed PG	The autoclave method	150	3	28.42	Gao et at.^18^
Washed PG	Microwave irradiation	100	1	12.3	Feng et al.^51^
PG and EMR	Microwave-assisted aqueous alcohol	100	3	39	This study

### The application of modified α-CSHWs

**Figure 13:**
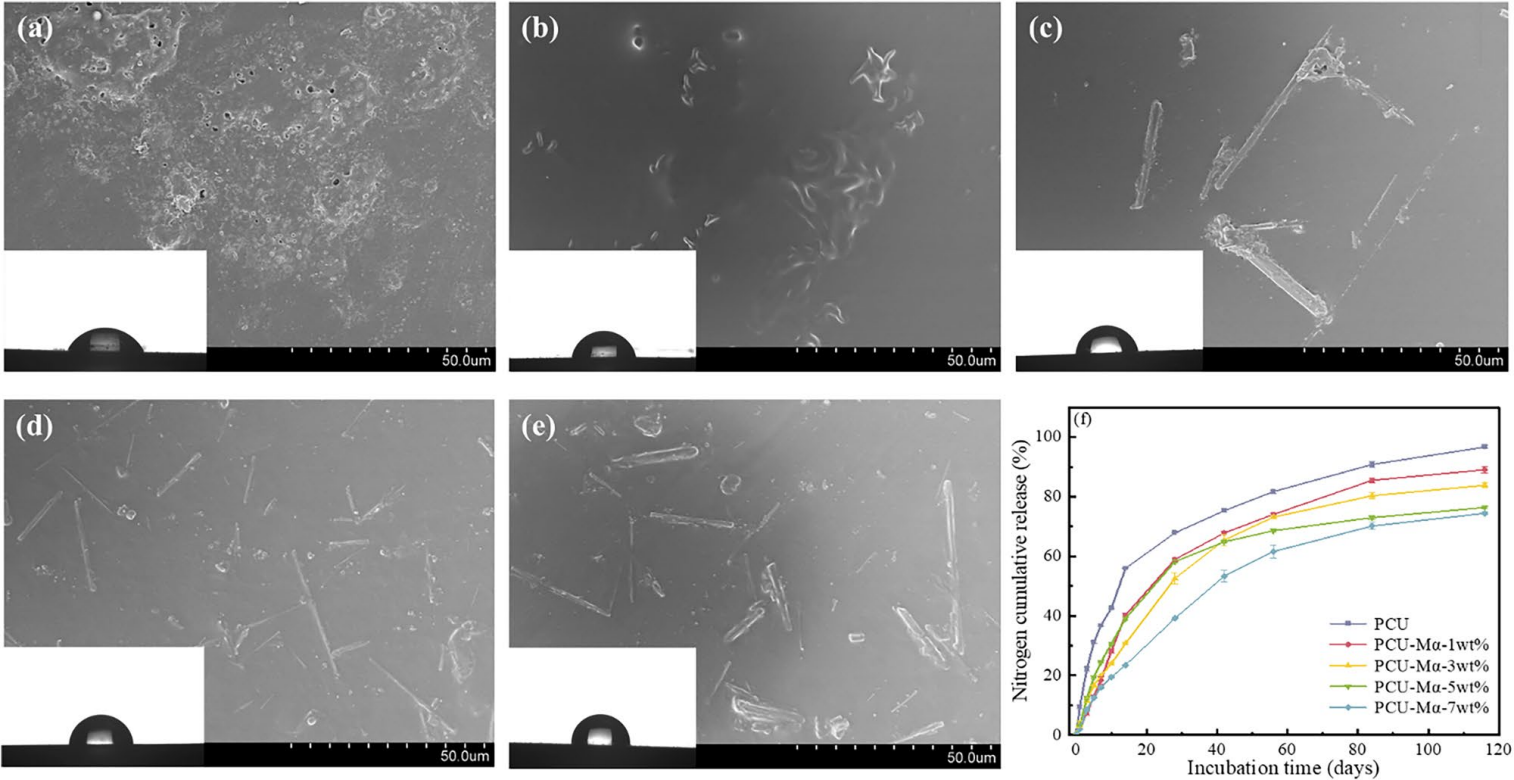
SEM and WCA images of the polyurethane films with different Mα dosage (%): (**a**) 0, (**b**) 1, (**c**) 3, (**d**) 5 and (**e**) 7%. (**f**) Nitrogen cumulative release of polyurethane-coated urea with different Mα dosage.

The microstructure of the polyurethane film was examined using scanning electron microscopy (SEM), as shown in Fig. 13a–e. Numerous pores were detected on the surface of the pure polyurethane film, which were detrimental to its controlled release performance. However, upon the addition of modified α-CSHWs (Mα), no pores were observed on the polyurethane film surface, indicating improved densification due to the effective integration of Mα within the polyurethane film. With increasing Mα content, the water contact angle of the polyurethane film increased. At Mα content levels of 0 wt%, 1 wt%, 3 wt%, 5 wt% and 7 wt%, the corresponding water contact angles of the polyurethane film were measured at 66.18°, 76.65°, 80.85°, 85.65° and 91.65°, respectively, indicating enhanced hydrophobicity due to Mα addition.

As shown in Fig. 13f, the initial nutrient release rate of the five prepared coated fertilizers was below 15%, and the nutrient release period exceeded 28 days, meeting the standards of ISO18644-2016 for controlled release fertilizers. The nutrient release period of pure polyurethane-coated urea (PCU) was 42 days. Upon incorporating Mα, the nutrient release periods for PCU-Mα-1wt% and PCU-Mα-3wt% were approximately 56 days, while PCU-Mα-5wt% and PCU-Mα-7wt% had nutrient release periods of approximately 116 days. Compared to pure polyurethane-coated fertilizer, the addition of Mα extended the nutrient release period and reduced the initial nutrient release rate.

## Conclusions

This study introduces, for the first time, the co-precipitation of impurities from electrolytic manganese residue (EMR) together with glycerol (PG) to synthesize α-CSHWs, followed by their application in controlled-release fertilizer. The influence of Mn^2+^ and NH_4_^+^ on the growth of α-CSHWs was investigated, and the mechanism of α-CSHW formation with the addition of EMR was elucidated through DFT calculations and analytical techniques such as FT-IR. Changes in the performance of the controlled-release fertilizer before and after the incorporation of modified whiskers were compared. Some meaningful findings are as follows:

1. The addition of EMR, through cooperative solidification with PG, forms insoluble impurities, reducing the soluble F^–^ and PO_4_^3–^ content in the whisker precursor. With an increase in the EMR content, the nucleation speed of the whiskers becomes faster. In particular, under 25wt% of EMR addition, whiskers with a length-to-diameter ratio of 39 can be obtained within 3 h.
2. Mechanistically, glycerol, Mn^2+^, and NH_4_^+^ provide favorable conditions for α-CSHWs nucleation. NH_4_^+^ promotes the radial growth of whiskers, thereby increasing the length-to-diameter ratio. F^–^ and PO_4_^3–^ ions, along with Mn^2+^, inhibit the radial growth of α-CSHWs. Therefore, utilizing an appropriate amount of EMR is a straightforward and feasible method for whisker synthesis.
3. The application of modified α-CSHWs in polyurethane-coated controlled release fertilizers showed that the inclusion of Mα significantly prolonged the nutrient release period of the coated fertilizers. In comparison to pure PCU, PCU-Mα at 5wt% and above extended the nutrient release period to 116 days. This presents a novel approach for the preparation of long-acting controlled-release fertilizers, although the mechanism of action of modified whiskers in controlled-release fertilizers requires further exploration.

### Supplementary Information

Supplementary Information.

## Data Availability

The datasets used and/or analyzed during the current study are available from the corresponding author upon reasonable request.
