# Supplementary material for: Synergistic preparation and application in PCU of α-calcium sulfate hemihydrate whiskers from phosphogypsum and electrolytic manganese residue
Source: Sci Rep. 2024 Mar 15;14:6260. doi: 10.1038/s41598-024-56817-5 (PMC10943202; doi:10.1038/s41598-024-56817-5)
Supplement: Supplementary file 1 — Supplementary Information. [file 41598_2024_56817_MOESM1_ESM.docx]

**Synergistic preparation and application in PCU of α-calcium sulfate hemihydrate whiskers from phosphogypsum and electrolytic manganese residue**

Ting Wang^a^, Xuan Ke^a^, Jia Li^a,^*, Ying Wang^a^, Weiwei Guan^a^, Xia Sha^a^, Chenjing Yang^a^, Tian C. Zhang^b^

^a^ Engineering Research Center for Heavy Metal Pollution Control of Hubei Province, College of Resources and Environmental Science, South-Central Minzu University, Wuhan 430074, China.

^b^ Civil & Environmental Engineering Department, College of Engineering, University of Nebraska-Lincoln, Omaha, NE, 68182, USA.

*Corresponding author

E-mail: jiajiali1982@aliyun.com

Tel: 18971601242

**1. Results and Discussion**

**1.1 XRD pattern of whisker precursor**

Figs. S1(a−b) show that the whisker precursors were composed of CaSO_4_·2H_2_O (PDF#01-70-0982), CaPO_3_(OH)·2H_2_O (PDF#00-11-0293), SiO_2_ (PDF#01-79-1906), CaF_2_(PDF#96-900-7063), MnF_2_(PDF#01-71-1832) and Mn_5_(OH)_4_(PO_4_)_2_(PDF#01-70-0516). With an increase in EMR dosage, the diffraction peak intensity of MnF_2_ gradually decreased. Fig. S1(c) reflects that the presence of impurities such as CaPO_3_(OH)·2H_2_O and Ca_5_F(PO_4_)_3_ in PG. The results indicate that F^-^ and PO_4_^3-^ in PG react with Mn^2+^ and Ca^2+^ in EMR during the mixed ball milling process, forming precipitates like MnF_2_, CaF_2_ and Mn_5_(OH)_4_(PO_4_)_2_. This leads to a reduction in the impurity content in the whisker precursor after the addition of EMR. The main equations involved are as followings:

$${Mn}^{2+}+2F^{-}\to{MnF}_{2}\boldsymbol{\downarrow}(1)$$

$${Ca}^{2+}+2F^{-}\to{CaF}_{2}\boldsymbol{\downarrow}(2)$$

$${3Ca}^{2+}+2\mathrm{PO}_{4}^{3-}\to\mathrm{Ca}_{3}{{(PO}_{4})}_{2}\boldsymbol{\downarrow}(3)$$

$${5Mn}^{2+}+2\mathrm{PO}_{4}^{3-}+4\mathrm{OH}^{-}\to\mathrm{Mn}_{5}{(OH)}_{4}{{(PO}_{4})}_{2}\boldsymbol{\downarrow}(4)$$

**
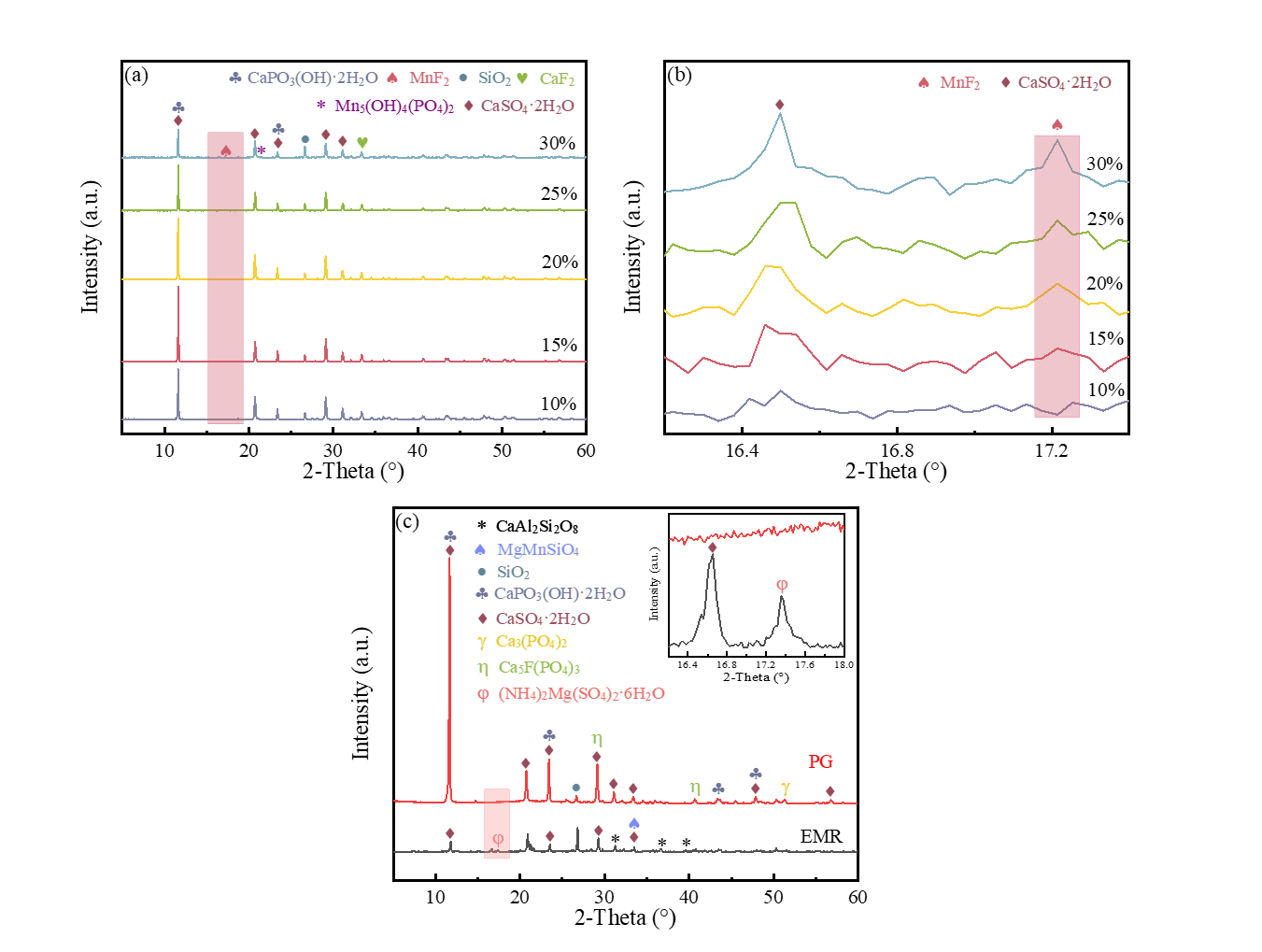
**

**Figure S1.** XRD patterns of the whisker precursors by different EMR dosage :(a) and (b), (c) XRD patterns of PG and EMR.

**1.2 Morphology of prepared CSHWs**

From Fig. S2, it is evident that, after a 2-h reaction, the whiskers generated consist of dihydrate calcium sulfate whiskers and small-sized blocks of SiO_2_. Point scanning reveals the presence of impurities remaining on the whisker surface, with SiO_2_ being one of them. However, due to the relatively low content of introduced Mn^2+^ and NH_4_^+^, they were not detected.


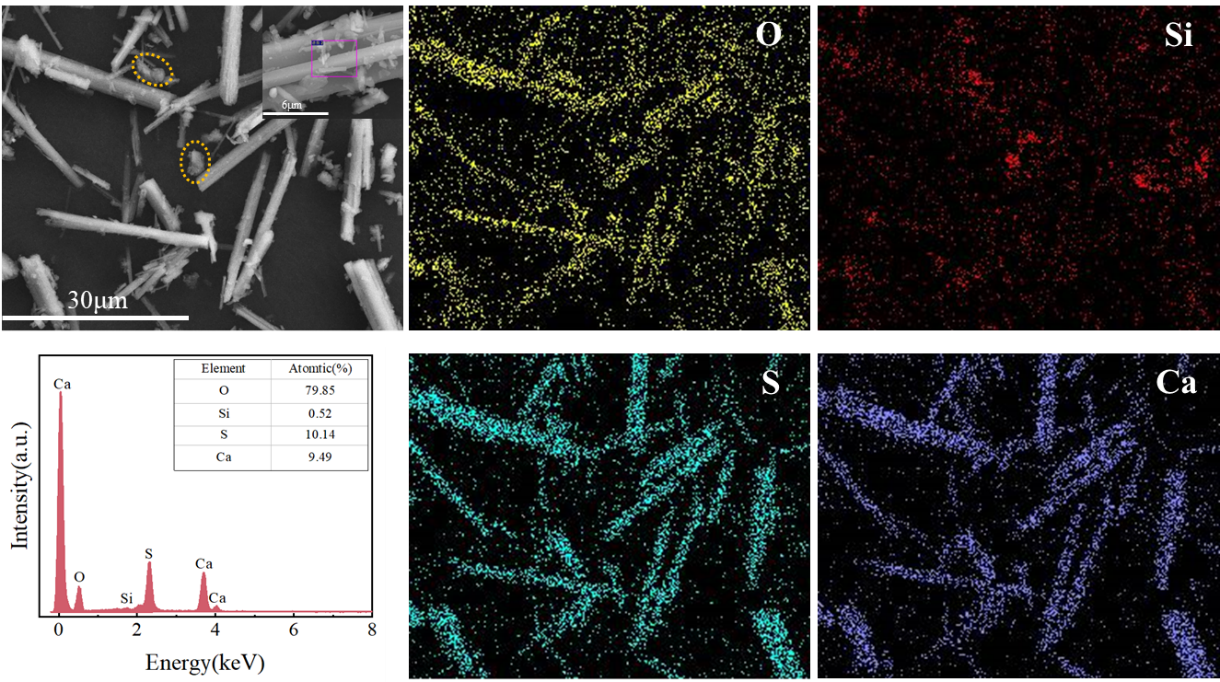


**Figure S2.** SEM-EDS images of the α-CSHWs synthesized in 2 h with 30% EMR dosage.

**1.3 Environmental implications**

Based on the solid waste leaching toxicity method (China HJ557–2010), leaching toxicity tests were conducted on the raw materials–EMR, PG, and the whiskers prepared within 2 hours. The results are presented in Table S1. Comparative analysis with GB 5085.3-2007 indicates that the ion concentrations in both the raw materials and the prepared whiskers remain below the specified limits.

**Table S1. Result of leaching toxicity tests (mg·L^-1^)**

| Element species | Mn | Cu | Zn | Cd | Pb | As | Cr | Ni |
| --- | --- | --- | --- | --- | --- | --- | --- | --- |
| GB 5085.3-2007 | — | 100 | 100 | 1 | 5 | 5 | 15 | 5 |
| PG | 1.88 | 0.171 | 1.087 | 0.006 | 0 | 0.136 | 0.006 | 0.235 |
| EMR | 2730.92 | 0 | 0.076 | 0 | 0 | 0.067 | 0 | 1.305 |
| Whisker - 0%EMR | 1.18 | 0.025 | 0.085 | 0.001 | 0 | 0.065 | 0 | 0.003 |
| Whisker - 10%EMR | 28.36 | 0.017 | 0.073 | 0 | 0 | 0.041 | 0 | 0.010 |
| Whisker - 15%EMR | 46.88 | 0 | 0.061 | 0 | 0 | 0.033 | 0 | 0.017 |
| Whisker - 20%EMR | 58.13 | 0 | 0.016 | 0 | 0 | 0.012 | 0 | 0.018 |
| Whisker - 25%EMR | 76.34 | 0 | 0 | 0 | 0 | 0 | 0 | 0.021 |
| Whisker - 30%EMR | 82.65 | 0 | 0 | 0 | 0 | 0 | 0 | 0.026 |

**
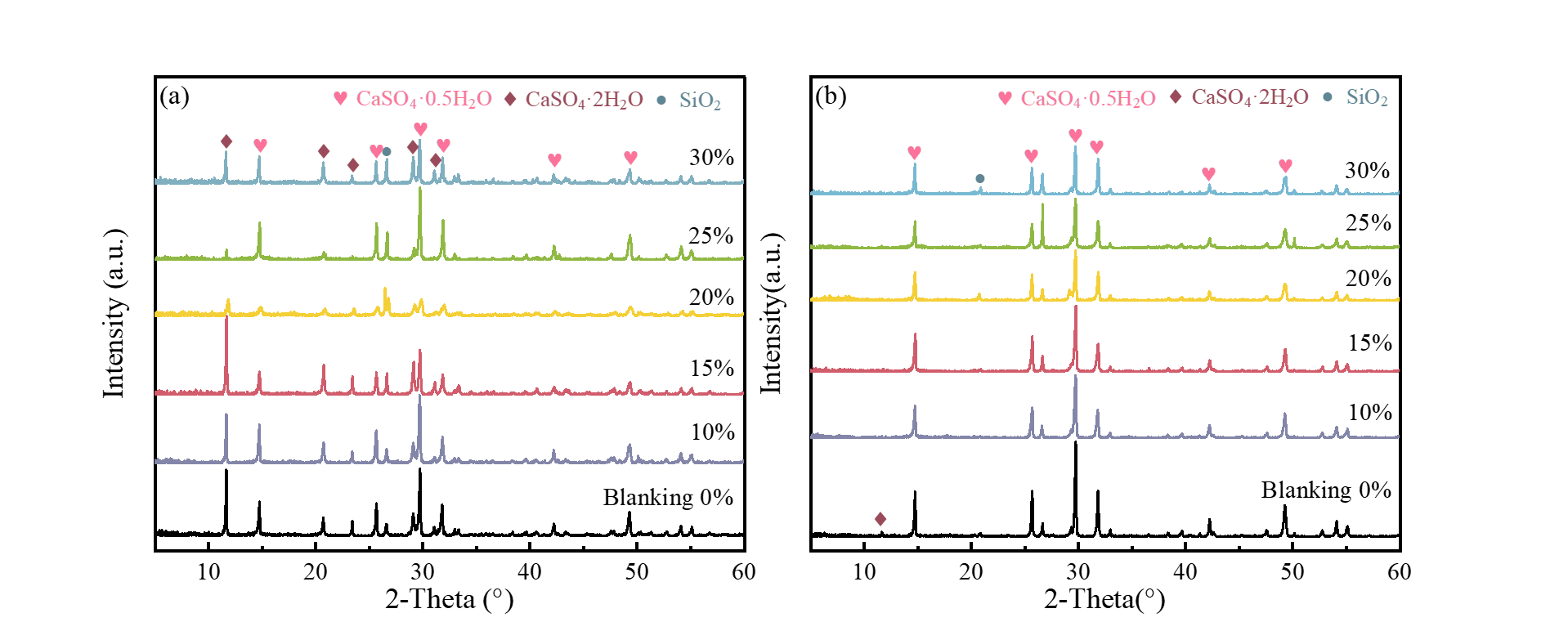
**

**Figure S3.** XRD patterns of the samples synthesized by different EMR dosage with different time (a) 1 h, (b)3h
